# Anchored fallopian tube through the drain tube: A rare case report

**DOI:** 10.1002/ccr3.5917

**Published:** 2022-05-27

**Authors:** Prezma Shrestha, Asma Kunwar, Yasoda Rijal, Susan Aryal, Yagya Raj Adhikari, Shiva Lal Bhattarai, Ashmita Gautam, Suniti Rawal

**Affiliations:** ^1^ Department of Obstetrics and Gynecology Tribhuvan University Teaching Hospital Kathmandu Nepal; ^2^ Maharajgunj Medical Campus Institute of Medicine Kathmandu Nepal

**Keywords:** drain tube, ectopic pregnancy, fallopian tube, salpingectomy

## Abstract

Anchored fallopian tube through the drain tube is rare. We present a case of a 27‐year‐old female patient who underwent right salpingectomy with the fenestrated drain tube in the pelvic cavity. Postoperatively, the drain could not be removed. Laparotomy revealed the left fallopian tube entering through the fenestration of the drain tube.

## Abstract 2

Anchored fallopian tube through the drain tube is a rare postoperative complication. This differential should be kept in mind if the drain tube in the pelvic cavity cannot be removed post‐salpingectomy.

## 1 INTRODUCTION

Closed suction drains are widely used in most surgical procedure to drain serosanguineous fluid from the abdominal and pelvic cavity. Ruptured ectopic pregnancy is one of the common gynecological conditions where the pelvic drain is used after surgical procedure. Most surgical drains are removed without any difficulties. Retained intraperitoneal drain is rare in the immediate postoperative period.^1^, ^2^ We present a peculiar case of stuck pelvic drain tube in which the fallopian tube entered through one fenestration and escaped through adjacent fenestration of the drain tube.

## 2 CASE DESCRIPTION

A 27‐year‐old female patient, G3P1A1, presented to the emergency department with the complaint of acute onset lower abdominal pain of one‐day duration, which was continuous, non‐radiating, associated with nausea and symptoms of anemia like dizziness and shortness of breath. There was no history of fever or a symptom suggestive of PID. Her last menstrual period (LMP) was 43 days back. She had regular cycles with flow of 4–5 days without dysmenorrhea. On examination, the patient looked pale and distressed. Her BP (blood pressure) was 90/60 with the pulse rate of 98 bpm (beats per minute). The abdomen was distended and tender, and cervical motion tenderness was present on per vaginal examination. Urine pregnancy test was positive. Her hemoglobin count was 6.9 gm%, PCV (packed cell volume) was 20.3, and WBC (white blood cell) count was 13 000/cmm. USG (ultrasonography) revealed an empty uterine cavity, mixed echoic focus measuring approximately 30 × 20mm in the right ampulla without internal vascularity and free fluid in the dependent part of the peritoneal cavity. On aspiration, 5 ml of frank blood was obtained. With the diagnosis of ruptured ectopic pregnancy, emergency laparotomy was done which revealed 32 × 25 mm of swelling in the right ampulla of the fallopian tube. Rent of 1 cm was noted in the ampulla with active oozing. On cut section, products of conception like material (gestational sac) seen. Around 3 L of blood was suctioned along with ongoing blood transfusion. The right fallopian tube was resected and peritoneal lavage was completed following which pelvic drain was placed.

**FIGURE 1:**
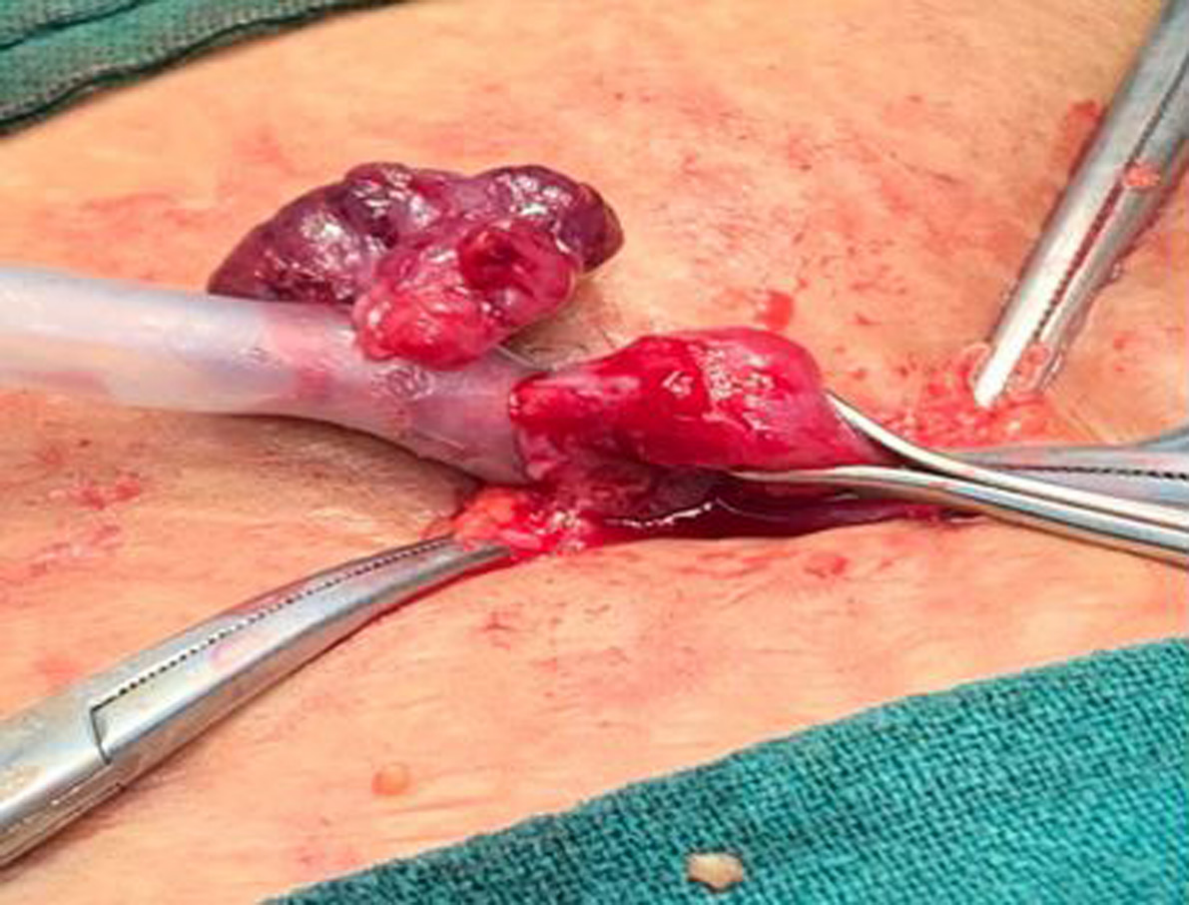
Fallopian tube entering through one fenestration of the drain tube and exiting through another fenestration

Postoperatively, the patient was stable hemodynamically. The pelvic drain contained serosanguineous fluid measuring 400, 300, 200, 150, and 80 ml in each consecutive days. On the fifth postoperative day, drain could not be removed. She had no history of fever and was passing stool and flatus. Chest X‐ray was normal with no gas under diaphragm. Ultrasound revealed drain tube in the pelvic cavity with minimal interloop ascites. With the provisional diagnosis of stuck drain, surgery was planned. Skin incision of around 5 cm was given, peritoneum was opened and the drain tube was exposed. The left fallopian tube was found to be anchored inside the drain tube with the fallopian tube entering from one eye and exiting through adjacent eye (Figure 1). The definitive diagnosis of stuck drain due to anchored fallopian tube was made intraoperatively. The drain was cut longitudinally from one eye to another and the stuck left fallopian tube was carefully released. On examination, the fallopian tube was pink and healthy. The drain was removed and abdomen was closed. The patient was stable postoperatively. After 3 months of surgery, she had her menstruation. She visited for a follow‐up after one year for preconception counseling.

## 3 DISCUSSION

Anchored tube through the drain as a postoperative complication is a rare condition. Based on the review of the literature, this is the first case of its kind to be reported. However, other complications of drain getting intertwined with intra‐abdominal structures, bleeding, intestinal loop strangulation, or site evisceration have been reported.^3^, ^4^ Some cases may not have been published for the fear of medico‐legal concerns.^5^, ^6^

Complications like bowel loop and omentum being stuck in the drain tube and formation of Richter type hernia after loop of bowel being caught in knotted drain tube have been previously reported.^3^, ^5^, ^7^ In this case, however, fallopian tube was anchored within the drain tube following right salpingectomy. Any delay in the diagnosis and intervention could have led to strangulation and ischemic necrosis of the fallopian tube, which was avoided in this case.

## 4 CONCLUSION

By reporting this case, we aim to highlight the possibility of entry of abdominal contents through the eye of drain tube as the potential cause of stuck drain following any kind of surgical procedures. Diagnosis of stuck drain is usually clinical when the patient complains of consistent pain on the drain site along with difficulty in its removal as most of the surgical drains are easily removed. Early intervention is necessary to avoid risks of complications including trauma and necrosis of the fallopian tube. Definitive diagnosis is usually only possible intraoperatively.

## AUTHOR CONTRIBUTIONS

Suniti Rawal (SR) and Prezma Shrestha(PS) involved in study concept, data collection, and surgical therapy for the patient. Prezma Shrestha (PS), Asma Kunwar (AK), and Yasoda Rijal (YR) involved in writing—original draft preparation. Susan Aryal (SA), Yagya Raj Adhikari (YRA), Shiva Lal Bhattarai (SLB), and Ashmita Gautam (AG) involved in editing and writing. SR and PS served as senior author and manuscript reviewer. All the authors read and approved the manuscript.

## CONFLICT OF INTEREST

None to declare.

## ETHICS APPROVAL

Not required.

## CONSENT

Written informed consent was obtained from the patient and her husband for the publication of this case report and accompanying images. A copy of the written consent is available for review by the Editor‐in‐Chief of this journal on request.

## Data Availability

All the necessary data and materials are within the manuscript.
